# Large Neutral Amino Acids (LNAAs) Supplementation Improves Neuropsychological Performances in Adult Patients with Phenylketonuria

**DOI:** 10.3390/nu12041092

**Published:** 2020-04-15

**Authors:** Iris Scala, Maria Pia Riccio, Maria Marino, Carmela Bravaccio, Giancarlo Parenti, Pietro Strisciuglio

**Affiliations:** 1 Department of Maternal and Child Health, Federico II University Hospital, 80131 Naples, Italy; carmela.bravaccio@unina.it (C.B.); parenti@unina.it (G.P.); 2 Department of Translational Medical Sciences, Section of Pediatrics, Federico II University, 80131 Naples, Italy; maria.marino06@gmail.com (M.M.); pietro.strisciuglio@unina.it (P.S.); 3 Telethon Institute of Genetics and Medicine (TIGEM), 80078 Pozzuoli (NA), Italy

**Keywords:** phenylketonuria, large neutral amino acids, treatment, phenylalanine, tyrosine, psychometric performance, psychometric tests, executive functions, cognition

## Abstract

Phenylketonuria is an inborn error of phenylalanine (Phe) metabolism diagnosed by newborn screening and treated early with diet. Although diet prevents intellectual disability, patients often show impairment of executive functions, working memory, sustained attention, and cognitive flexibility. Large neutral amino acids (LNAAs) have been proposed as a dietary supplement for PKU adults. Few studies show that LNAAs may help in improving metabolic control as well as cognitive functions. In this study, 10 adult PKU patients with poor metabolic control were treated for 12 months with LNAAs (MovisCom, 0.8–1 g/kg/day) and underwent Phe and Tyrosine (Tyr) monitoring monthly. Neuropsychological assessment was performed at T0, T+3, and T+12 months by using the American Psychological General Well-Being Index, the Wisconsin Card Sorting Test, the Test of Attentional Performance, and the 9-Hole Peg Test. No change in plasma Phe levels was observed during LNAAs supplementation, while Tyr levels significantly improved during LNAAs supplementation (*p* = 0.03). Psychometric tests showed an improvement of distress and well-being rates, of executive functions, attention, and vigilance, whereas no difference was noted regarding hand dexterity. This study adds evidence of the advantage of LNAAs supplementation in improving cognitive functions and well-being in patients with PKU with poor metabolic control.

## 1. Introduction

Phenylketonuria (PKU; MIM 261600) is an autosomal recessive disorder caused by the deficiency of phenylalanine hydroxylase (PAH, EC 1.14.16.1), the hepatic enzyme that converts phenylalanine (Phe) to tyrosine (Tyr), using tetrahydrobiopterin (BH4) as coenzyme. More than 500 mutations of the PAH gene have been described so far (http://www.pahdb.mcgill.ca; http://www.biopku.org). PKU is classified into classic PKU (cPKU), moderate PKU (moPKU), and mild PKU (mPKU) according to plasma Phe levels at diagnosis and tolerance, defined as the highest Phe intake able to keep blood Phe levels within the safe range [1]. Hyperphenylalaninemia is also divided in mild hyperphenylalaninemia (HPA)-gray zone, requiring dietary protein restriction, and mild hyperphenylalaninemia where no treatment is required as Phe levels are below 360 μmol/L [1]. If untreated, PKU leads to neurological abnormalities, while early treatment with a Phe-restricted diet prevents brain damage and results in almost normal neurological development [2]. However, although intelligence is within the normal range, patients with PKU often show executive function (EF) impairment compared to healthy controls [3] as well as deficit of working memory [4,5], inhibitory control [4,6,7], sustained attention [8], cognitive flexibility [7,9], verbal fluency [5,10], and planning [8,11,12].

The disturbance of Phe metabolism causes the depletion of Tyr, a precursor of the neurotransmitter dopamine. In addition, Phe accumulates and competes with Tyr and tryptophan, a precursor of serotonin, to cross the blood–brain barrier (BBB). As a consequence, the main effect of this disorder on the nervous system is a deficiency of dopamine and serotonin neurotransmitters [13].

The mainstay of PKU treatment is still a low protein diet, supplemented with Phe-free amino acid formulas and vitamins. However, this treatment is often complicated by psychological discomfort and reduced compliance to the diet after the first 4–5 years of life. Initial reports supported the possibility to loosen the diet during adulthood; however, as defects of executive functions and brain hypomyelination may occur in patients with poor metabolic control [14,15], a life-long dietary therapy is recommended. Hence, alternative therapies have been developed such as sapropterin (Kuvan, Biomarin) for PKU patients responsive to BH4, and enzyme substitution therapy (Pegvaliase, Biomarin) for adult patients. Gene therapy is under development.

Large neutral amino acids (LNAAs) supplementation has been developed for PKU patients with a loosen diet and poor compliance to diet. Phe and other neutral amino acids (LNAAs: Tyr, tryptophane, threonine, methionine, valine, isoleucine, leucine, and histidine) share the same transporter in the brain and in the intestinal mucosa. In healthy subjects, all these amino acids, except Tyr, are essential amino acids. In patients with PKU, also Tyr becomes an essential amino acid. The direct effect of high brain concentrations of Phe and low concentrations of LNAAs is probably the main cause of an altered brain development and functioning in patients with PKU [16]. The possible targets of the treatment with LNAAs include reducing brain Phe levels, reducing Phe plasma concentrations, and increasing the synthesis of neurotransmitters in the brain and/or the increase of non-Phe LNAAs in the brain [17].

LNAAs transport across BBB was studied for the first time in mice in 1976 [18]. Since then, different combinations of LNAAs have been produced with increasing qualitative and quantitative characteristics. In a first clinical trial, the supplementation with Tyr (160 mg/day) in PKU patients was effective in increasing attention span and neurotransmitter metabolites concentration in the brain [19]. In a subsequent study, no improvement in neuropsychological tests was achieved by using Tyr at the lower dose of 100 mg/day [20]. Ten years later, the open label clinical study by Matalon and colleagues [21] documented the reduction of blood Phe levels in mice and humans with PKU by using a mixture of LNAAs as a dietary supplement. This study enrolled 11 patients. Eight patients received 0.5 g/kg/day and three patients 1 g/kg/day of LNAAs (NeoPhe) divided into three daily doses to be taken before meals. Phe levels were determined at baseline and after 1 week of LNAAs supplementation. The blood concentration of Phe in the 8 patients taking 0.5 g/kg/day of LNAAs was reduced by 52%; in the 3 patients taking 1 g/kg/day of LNAAs, Phe levels declined by 55% from the baseline. The following double-blind placebo-controlled study [22] confirmed a decline of 39% of plasma Phe levels in patients with PKU treated with LNAAs (0.5 g/kg/day) for 2 weeks. In the same year, a prospective double blind cross-over study consisting in four 2-weeks phases with LNAAs supplementation (250 mg/day) or placebo, with or without the patient’s usual medical formula, evaluated plasma and brain Phe by magnetic resonance spectroscopy and positron emission tomography as well as executive functions in 16 cPKU patients. The authors showed a reduction of brain Phe and an improvement of executive functions, such as verbal generativity, cognitive flexibility, and self-monitoring. However, no lowering of plasma Phe could be observed [23]. A subsequent study on 9 adult PKU patients consisting of four 4-weeks phases evaluated the effects on blood Phe, melatonin, and dopamine of a supplementation with LNAAs (0.5 g/kg/day, PheBloc, Applied Nutrition, Cedar Knolls, NJ, USA) with or without BH4. No change in plasma Phe levels was demonstrated on LNAAs supplementation, while a positive synergistic effect on serum melatonin was observed in combination with BH4 therapy [24]. More recently, the study by Concolino and colleagues [25] dosed 2-h post prandial Phe and Tyr levels in 12 PKU patients supplemented with 0.5 g/kg/day LNAAs for 4 weeks. The study confirmed a 58% reduction of Phe from baseline and a significant raise of Tyr levels. Finally, a significant increase of Tyr levels was found after a 12-month period of LNAAs supplementation in 12 adult PKU patients [26].

Taken together, literature data published so far on the effect of LNAAs supplementation in PKU are still scarce. Studies differ in LNAAs dose, therapy duration, and endpoints. However, the possible role of LNAAs in improving neurotransmitter derangement in PKU and, subsequently, neuropsychological performances deserve further attention and additional evidence. In the present study, we aimed at evaluating the effect of a long-term (12 months) high dose LNAAs supplementation (0.8–1 g/kg/day) on plasma Phe and Tyr levels, on executive function, vigilance, attention, and on the perceived patients’ quality of life.

## 2. Materials and Methods

### 2.1. Patients

Ten adult patients (6 F/4 M, mean age 23.6 ± 4.5 years; range 18–32 years) with PAH deficiency were recruited at the Department of Maternal and Child Health, Federico II University Hospital, after Ethical Committee approval and signed informed consent (Ethical Committee Approval n. 30/15). The study was carried out in accordance with the Declaration of Helsinki and ICH/GCP.

Inclusion criteria were age ≥18 years, early dietary intervention after neonatal diagnosis, PKU phenotype requiring dietary treatment and amino acid supplementation, complete PAH genotyping, normal intelligence (IQ > 80), poor metabolic control during the last 12 months, adherence to study protocol after informed consent signature. 

Exclusion criteria were age <18 years, subjects with primary BH4 deficiency or with HPA not requiring dietary restriction, incomplete PAH genotyping, concomitant chronic diseases, patients not able to adhere to study procedure.

Patients were phenotypically classified according to neonatal Phe blood value before dietary treatment was started and to Phe tolerance, as determined at 5 years of age (historical tolerance). Four different phenotypes were identified: (1) classic PKU (cPKU; Phe > 1200 μmol/L; tolerance ≤ 350 mg/day), (2) moderate PKU (moPKU; Phe 900–1200 μmol/L; tolerance 350–400 mg/day), (3) mild PKU (mPKU; Phe 600–900 μmol/L; tolerance 400–600 mg/day), and (4) mild HPA-gray zone (Phe 360–600 μmol/L; tolerance > 600 mg/day), requiring dietary protein restriction [1]. When a discrepancy was noted between pre-treatment blood Phe levels and Phe tolerance, patients were classified according to Phe tolerance [27,28]. Table 1 reports age, sex, genotype, phenotype, and the prescribed LNAAs dose expressed in g/kg/day.

### 2.2. Supplementation with LNAAs

Patients were supplemented for 12 months with LNAAs (PheLNAA, MovisCom) at the dosage of 0.8–1 g/kg/day divided in 3 daily doses at main meals, with complete replacement of the previous non-LNAA formula. The nutritional facts of the LNAAs formula used in this study is reported in Table 2. Patients were asked to continue their diet without changing food habits, whereas the previous amino acid and vitamin supplements were withdrawn and replaced with PheLNAA.

Each patient underwent Phe, Tyr, Phe/Tyr ratio monitoring monthly. A 3-days meal diary was filled-in before each blood withdrawal. 

### 2.3. Plasma Phe and Tyr Determinations

Median plasma Phe (μmol/L), Tyr (μmol/L), and Phe/Tyr ratio were calculated from plasma Phe and Tyr values obtained during the 12 months preceding the inclusion into the study and compared with median plasma Phe and Tyr dosed during the study.

Phe, Tyr, and Phe/tyr ratio were dosed by HPLC (Agilent Technologies 1200 Series LC System).

### 2.4. Neuropsychological Assessment

Neuropsychological assessment was performed at T0, T+3, and T+12 months by using the American Psychological General Well-Being Index (PGWBI) for the evaluation of the perceived level of quality of life [29,30], the Wisconsin Card Sorting Test (WCST) for the evaluation of executive functions [31], the Test of Attentional Performance (TAP test) for the evaluation of alertness and concentration [32], and the 9-Hole Peg Test (HPG test) for the evaluation of digital dexterity [33].

### 2.5. Psychological General Well-Being Index (PGWBI)

The Psychological General Well-Being Index (PGWBI) is a self-report questionnaire of 22 items organized in six subscales: Anxiety, Depression, Well-being, Self-control, Health, and Vitality. Responses for each item are evaluated on a six-point Likert scale ranging from 0 to 5. Higher scores indicate better well-being. The subscales sum provides a global index score for subjective well-being (range 0–110). Considering “distress” as the reverse of well-being, a global score <60 suggests a “Severe Distress”; from 60 to 72 a “Moderate Distress”; and >72 “No Distress” category [30].

### 2.6. Wisconsin Card Sorting Test (WCST)

The Wisconsin Card Sorting Test (WCST) is a neuropsychological tool to test abstract reasoning and the ability to change cognitive strategies when environmental circumstances change. The test evaluates executive functions and flexibility. The WCST explores the following frontal lobe functions: strategic planning, organized searching, utilizing environmental feedback to shift cognitive sets, directing behavior to achieve a goal, and modulating impulsive responding. The test is used with subjects aged from 6.5 years to 89 years. In the present study, the following test variables were evaluated: (1) perseverative errors, reflecting problems in switching from one rule to another; (2) non-perseverative errors, associated with absent-mindedness of attention; (3) total of all errors, which explores functional status of cognitive flexibility and attention; (4) total amount of categories completed, which tests cognitive processing success; (5) total amount of cards to complete the first category, which reflects the efficiency/speed of identifying the first classification algorithm.

### 2.7. Test of Attentional Performance (TAP Test)

The Test of Attentional Performance (TAP test) is a computer-based test exploring eight attention domains such as spatial attention, sustained attention, vigilance, and working memory. The test quantifies the speed (mean, median and standard deviation, msec) and the accuracy of the answers (errors, omissions). The computer-based test to assess vigilance gives mean, median and SD in ms for the first section, the second section, and the total time to complete the tasks. 

### 2.8. 9-Hole Peg Test (HPG)

The 9HPT is a brief, standardized, quantitative test of upper extremity function. It explores fine motor function and eye-hand coordination. Participants performed two consecutive rounds both with the dominant and non-dominant hand. The score for the HPT was an average of the 2 rounds for each hand.

### 2.9. Statistical Analysis

Normality of continuous data was assessed by the Levene test. The Student’s *t*-test was used to compare means and the Mann–Whitney U-test to compare medians. Pearson’s correlation coefficient (*r*) was used to measure the strength of the association between two variables. A two-tailed *p*-value <0.05 was assumed as statistical significant. Statistical analysis was performed by SPSS 17.0 software.

## 3. Results

### 3.1. Phenotypic Classification

Ten adult PKU patients (6 females/4 males) completed the study: 7 patients were classified as cPKU, 1 patient as moPKU and 2 patients as mPKU. In patient #2, the neonatal pre-treatment Phe level (1200 μmol/L) was discordant with the observed historical tolerance (450 mg Phe/day) and was classified as mPKU according to tolerance, as detailed in the Methods section. All patients received complete genotyping (Table 1).

### 3.2. Phe, Tyr, and Phe/Tyr Ratio

All patients received a daily LNAAs dose of 0.8–1 g/kg/day divided in three administrations at main meals. No significant change in plasma Phe levels was observed during the LNAAs supplementation except for patient #4 who had a significant raise of Phe levels due to worsening of dietary compliance. Hence, in adult patients with poor compliance to dietary treatment, we could not demonstrate a reduction of plasma Phe and an improvement of the metabolic control under LNAAs supplementation.

Plasma Tyr levels significantly raised in 9/10 patients and the Phe/Tyr ratio significantly declined in 5/10 patients (Table 3). Considering the PKU patients as a group, Tyr levels significantly improved during LNAAs supplementation (*p* = 0.03).

### 3.3. Psychometric Evaluation

#### 3.3.1. Psychological General Well-Being Index (PGWBI)

**Figure 1:**
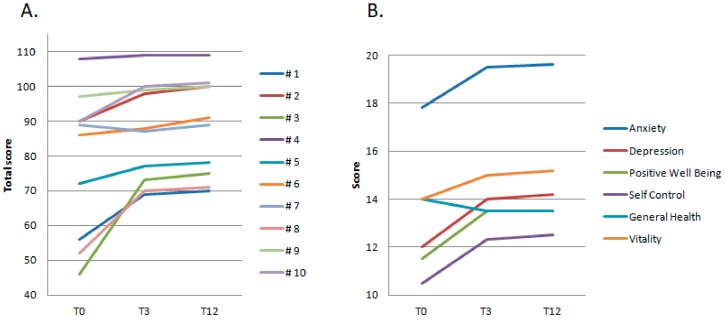
Psychological General Well-Being Index (PGWBI). (**A**) Total score index for each patient (#1–10) at T0 and after 3 and 12 months of LNAAs supplementation (T3; T12); (**B**) Mean values of the PGWBI subscales at T0, T3, and T12.

All patients completed the PGWBI health related quality of life test (Figure 1). The descriptive analysis showed that 3/10 patients presented at T0 with severe distress (total score 56, 46, and 52, respectively), 1/10 patients with a borderline-mild distress (total score 72), and 6/10 patients with positive well-being. After 3 months of LNAAs supplementation (T3), all patients showed an improvement of their general well-being, confirmed at the end of the study, despite the difference was not statistically significant (mean score ± SD: T0 78 ± 21; T3 87 ± 14; T12 88.4 ± 14). The improvement was more evident in those patients showing severe distress scores at T0: patient #1 improved his distress from severe to mild-moderate (Total score: T0 56, T3 69, T12 70) with better scores in the anxiety, self-control, general health, and vitality subscales; patient #3 improved from a severe distress to a positive well-being condition (Total score: T0 46, T3 73, T12 75) with relevant improvement in the depression, positive well-being, self-control, and vitality subscales; patient #8 improved from a severe to a mild distress condition (Total score: T0 52, T3 70, T12 71) with better rating in the anxiety, depression, positive well-being, and self-control subscales. Patient #5, presenting at T0 with a borderline-mild distress score (T0: 72), improved his perceived general well-being to a normal score (T3: 77, T12: 78) mainly due to better anxiety and vitality scores.

#### 3.3.2. Wisconsin Card Sorting Test (WCST)

PKU patients were able to complete all six card classification categories (6/6) as proposed by the WCST (n. of categories completed) at T0, T3, and T12, except patient #2 that was able to complete 5 out of 6 categories at T0 and 6 out of 6 categories after 3 and 12 months of LNAAs supplementation (T3, T12). In order to accomplish the test, at T3 and T12, PKU patients needed less cards compared to T0 (83.5 and 82.8 vs. 93.6 out of 128 available cards). The total number of errors and the percent of errors significantly decreased during the LNAAs supplementation along with a significant improvement of the performance centile (*p* < 0.05). A tendency toward significancy was observed in the areas of perseverative errors (*p* = 0.06) and the % of perseverative errors (*p* = 0.08), reflecting a more flexible and adaptive behavior regarding the task. The significant reduction of non-perseverative errors (*p* = 0.03) and the % of non-perseverative errors (*p* = 0.001) reflected a reduction of the random responses during the task. Finally, the % of conceptual level responses significantly increased (*p* = 0.03), indicating that the reduction of errors was obtained for a raise of intentional right responses excluding random “rights” (Table 4).

#### 3.3.3. Test of Attentional Performance (TAP test)

Vigilance and sustained attention were explored by the TAP test. Vigilance improved in all patients. The time to complete the total tasks, expressed in ms, significantly improved after 3 and 12 months of LNAAs supplementation (Mean ± SD: T0 706 ± 82 ms, T3 614 ± 64 ms; T12 609 ± 60 ms, *p* < 0.05). The total time (ms) to complete the tasks proposed to assess sustained attention improved after 3 and 12 months of LNAAs supplementation (Mean ± SD: T0 727 ± 147 ms, T3 597 ± 106 ms, T12 589 ± 98 ms; *p* < 0.01). In addition, the number of errors throughout the test significantly decreased as well as the number of omissions (Table 5).

#### 3.3.4. 9-Hole Peg Test (HPG)

The fine motor function and eye-hand coordination were explored by the 9-Hole Peg test for hand dexterity. Each patient performed two consecutive rounds both with the dominant and non-dominant hand and the performance was expressed in seconds. No significant difference in hand dexterity was observed during LNAAs supplementation in the analyzed group (mean ± SD T0 23 ± 3, T3 21 ± 3.6, T12 21.4 ± 2 s, *p* > 0.05). Only patient #1 and #5 improved their speed in the HPG test: patient #1 improved the speed of 3 s at T3 and of 4 s at T12 compared to 20 s at T0; patient #5 improved the speed of 8 s at both T3 and T12 compared to 24 s at T0.

## 4. Discussion

PKU is a rare metabolic disorder affecting 1:10,000 live births. After the success of the newborn screening programs and the efficacy of early low-Phe dietary intervention in averting the risk of intellectual disability in children, the attention of the scientific community is increasingly shifting from childhood to adulthood. Indeed, adults living with PKU experience deep psychological discomfort and neurological symptoms such as attention deficit, irritability, anxiety, headaches, and worst executive functions compared to age-matched controls. In childhood as in adulthood, the follow-up programs have emphasized plasma Phe levels as the most important outcome measure, although this now appears reductive. Therefore, great efforts have been made to identify novel biomarkers besides Phe to monitor patients’ health, as well as neuropsychological measures susceptible of improvement. In addition, alternative pharmacological therapies have been developed such as Sapropterin and Pegvaliase (Biomarin).

The present study explored the effects of a medical formula enriched with LNAAs in 10 adult patients with PKU with a story of poor compliance to diet in the year before enrolment.

LNAAs formulas have been developed for PKU patients with poor compliance to diet. In this study, we choose to monitor plasma Phe and Tyr levels over a long time-period (12 months) and patients were asked to keep their dietary habits unchanged during the trial in order to obtain Phe and Tyr measures as realistic as possible. Under these conditions, we choose to supplement with the highest dose of LNAAs used so far in clinical trials (0.8–1 g/kg/day) [21,26] with the hope to achieve the best competition between dietary Phe and LNAAs at the level of the intestinal LAT transporter. Unfortunately, the present study failed to show a reduction of plasma Phe levels over the long period. This observation is consistent with previous studies [23,24] despite those studies using lower doses of LNAAs preparations (0.25 and 0.5 g/kg/day, respectively). Conversely, we could not replicate the data on plasma Phe from other studies [21,22,25] even if the LNAAs dose was as high as the dose used in the first report. In our opinion, this could be a consequence of the different study time-length. In previous studies, supplementation with LNAAs spanned from 1 to 4 weeks, a short period in which even patients poorly compliant to diet could better control their dietary Phe intake while knowing to be enrolled in a trial. Indeed, in the present study, the first plasma Phe value after the first month of Phe supplementation was lower by 23% to 50% from the baseline in 4 out of 10 patients. As a matter of fact, during the first weeks, those patients spontaneously reduced their dietary Phe amounts, as outlined by meals diaries. However, in all patients, Phe levels gradually raised during the following months as they went back to their previous dietary habits. This observation is in agreement with the data by Burlina and colleagues [26] which show a reduction of plasma Phe after the first 6-months of LNAAs supplementation but not after 12 months.

LNAAs formulas, compared to other amino acids mixtures, contain a high amount of Tyr and tryptophan per 100 g. During the study, we could observe a significant improvement of blood Tyr levels. Due to PAH deficiency, in PKU, Tyr becomes an essential amino acid. Tyr is the precursor of dopamine and norepinephrine, putatively involved in motor function, mood, arousal, attention, and anxiety [16]. In addition, the accumulation of Phe competes with the available Tyr and tryptophan (a precursor of serotonin) to cross the BBB. Therefore, the main effect of this disorder on the nervous system consists in a deficiency of dopamine and serotonin neurotransmitters.

In this study, the perceived level of quality of life, executive functions, vigilance, and attention span were evaluated by the Psychological General Well-Being Index (PGWBI), the Wisconsin Card Sorting Test (WCST), the TAP test, and the HPG test. The results showed an improvement of distress and well-being rates, of executive functions, sustained attention, and vigilance, whereas no difference was noted regarding hand dexterity. The improvement was observed after the first 3 months of LNAAs supplementation and was maintained at the end of the study. As Phe levels remained well above the desired levels in all patients, we can speculate that the difference in psychological performances may be due to the rise of LNAAs in the brain, including Tyr.

The present study has some limitations, including the low sample size and the lack of placebo. We also do not have data on the levels of neurotransmitter metabolites and we could not correlate the levels of plasma Phe and Tyr with the levels in the cerebrospinal fluid (CSF) nor the improvement of the psychological performance with the variation of neurotransmitters. We and others [26] suggest that the improvement of blood Tyr may be beneficial to attenuate the neurotransmitter imbalance in PKU, however we cannot exclude the opposite hypothesis, i.e., that a high dose of LNAAs may have a detrimental effect on Tyr transport across the BBB. Despite those limitations, these results add further evidence of the advantage of LNAAs supplementation in improving cognition and well-being in patients with PKU and poor metabolic control. In future studies, it would be interesting to correlate plasma amino acids and neurotransmitter metabolites with clinical data and to assess whether the cessation of LNAAs treatment would cause a decline of psychometric scores after initial improvements.

## Figures and Tables

**Table 1:** Age at enrolment, sex, genotype, phenylalanine (Phe) at diagnosis, historical tolerance, phenotype, and large neutral amino acids (LNAAs) supplementation of the enrolled subjects.

Subject *n*	Age (Years)	Sex	Genotype	Phe at Diagnosis (Μmol/L)	Historical Tolerance (mg Phe/day)	Phenotype	LNAA (g/kg/day)
1	27	F	R261Q °/ IVS10NT-11G>A	1870	350	cPKU	0.9
2	32	M	R261Q °/ P281L	1200	450	mPKU	0.8
3	24	F	R261Q°/ IVS10NT-11G>A	1500	350	cPKU	0.9
4	21	M	R243X/ IVS10NT-11G>A	2480	275	cPKU	0.8
5	20	M	R261Q°/ IVS10NT-11G>A	1694	340	cPKU	0.8
6	26	F	P281L/ W187X	1150	390	moPKU	0.8
7	27	F	R261Q °/ IVS10NT-11G>A	1815	265	cPKU	0.9
8	23	F	IVS10NT-11G>A/ IVS10NT-11G>A	1633	230	cPKU	0.8
9	18	M	R158Q °/ IVS10NT-11G>A	1200	330	cPKU	0.9
10	18	F	L48S °/ D222G	665	450	mPKU	0.8

The sign ° indicates mutations of the PAH gene known to have a predicted residual enzyme activity and to be responsive to BH4. Phe: phenylalanine; LNAAs: large neutral amino acids.

**Table 2:** Large neutral amino acids (LNAAs): nutritional facts per 100 g of product.

Energy	1546 kJ/365 kcal
Fat of which saturated fat	1 g 0.7 g
Carbohydrates of which sugars	12 g 12 g
Fibers	5 g
**Amino acids**	
L-tyrosine	12.4 g
L-Leucine	6.46 g
L-Lysine	3.76 g
L- Glutamine	2.32 g
L-proline	2.28 g
L-valine	2.2 g
L-isoleucine	2.2 g
L-tryptophan	2.2 g
L-threonine	2.08 g
L-arginine	1.84 g
Aspartate	1.56 g
L-histidine	0.8 g
L-methionine	0.64 g
**Vitamins**	
Vitamin C	66 mg
Vitamin E	13.2 mg
Niacin	13.2 mg
Pantothenic acid	5.78 mg
Vitamin B6	3.56 mg
Riboflavin	1.18 mg
Thiamine	1 mg
Vitamin A	858 mcg
Folic acid	495 μg
Biotin	99 μg
Vitamin K	74.6 μg
Vitamin D	10.24 μg
Vitamin B12	5.96 μg
**Minerals**	
Calcium	1056 mg
Chloride	916 mg
Phosphorus	816 mg
Magnesium	330 mg
Iron	13.2 mg
Sodium	<29.6 mg
**Other**	
Zinc	9.9 mg
Manganese	1.98 mg
Copper	1 mg
Iodine	132 μg
Molybdenum	59.4 μg
Selenium	49.5 μg
Chrome	26.4 μg
DHA	400 mg
Choline	429 mg
Inositol	82.5 mg

**Table 3:** Comparison of median plasma phenylalanine (Phe), Tyrosine (Tyr), and phenylalanine/ Tyrosine (Phe/Tyr) ratio before and during large neutral amino acids (LNAAs) supplementation.

Patients	Phe Pre-LNAAs *n* = 12	Phe on LNAAs *n* = 12	Tyr Pre-LNAAs *n* = 12	Tyr on LNAAs *n* = 12	Phe/Tyr Ratio Pre LNAAs *n* = 12	Phe/Tyr Ratio on LNAAs *n* = 12	Phe Level at T0, T3, and T12	Tyr Level at T0, T3, and T12
1	786	907	80	86	8.7	12.5	780; 890; 920	46; 115; 118
	(453–968)	(786–1028)	(38–99)	(54–118)	(8.1–18.7)	(9–15.8)		
2	907	1028	38	58 *	24	19 **	1068; 1080; 989	34; 78; 75
	(363–1089)	(847–1089)	(32–54)	(60–76)	(6–26)	(10–19)		
3	847	605	37	77 *	26	9.8 **	800; 550; 620	33, 140; 151
	(647–1082)	(423–1120)	(27.5–49)	(48–151)	(15–30	(4.6–17.7)		
4	937	1331 *	50	83 **	19	15	910; 1200; 1260	36; 88; 90
	(907–968)	(1028–1450)	(34–58)	(74–91)	(16–20)	(9.6–16)		
5	665	828	54	77 *	8.6	10	650; 800; 850	39; 95; 110
	(326–689)	(726–968)	(38–65)	(73–137)	(5–13.5)	(7.5–11.4)		
6	786	968	39	71 *	25	20	830; 800; 938	35; 73; 80
	(665–847)	(786–1089)	(30–50)	(60–80)	(16–29)	(17–21)		
7	907	877	45	70 **	20	13 **	920; 780; 805	36; 73; 79
	(689–968)	(726–1089)	(35–56)	(62–77)	(16–22)	(10–14)		
8	883	1056	41	52 *	21	19	900; 950; 890	33; 54; 60
	(665–1200)	(780–1260)	(31–49)	(46–63)	(18–28)	(16–23)		
9	810	948	65	238 **	11	3.3 **	956; 880; 920	57; 232; 240
	(480–1140)	(720–980)	(51–91)	(233–242)	(6–15)	(3–4)		
10	780	540	40	80 *	25	10 **	740; 439; 560	32; 87; 86
	(480–840)	(420–600)	(30–45)	(50–89)	(19–27)	(7–12)		

Biochemical data were collected during the 12 months before the enrollment into the study and during the following 12 months of LNAAs supplementation. Phe and Tyr levels are expressed in μmol/L. Data are reported as medians (10°–90° centile). * *p* < 0.05; ** *p* < 0.01. Columns 8 and 9 report, for each patient, plasma Phe and Tyr levels at T0, T3, and T12 when patients performed the psychometric evaluation.

**Table 4:** The Wisconsin Sorting Card Test results before and on LNAAs therapy.

Variables	T0 Raw Scores Mean (SD)	T3 Raw Scores Mean (SD)	T12 Raw Scores Mean (SD)	T0 Centile	T3 Centile	T12 Centile
Number of trials administered	93.6 (17.32)	83.5 (16)	82.8 (15)	--	--	
Total number correct	71.6 (6)	70.8 (11.4)	70 (11)	--	--	
Total number of errors	21.9 (12)	12.8 (6) *	12.4 (5.2) *	55 (33.6)	84 (20) *	86 (18) *
Percent errors	22 (8.5)	15 (4.5) *	15 (4) *	55 (33.6)	84 (20) *	87 (24) *
Perseverative responses	15.3 (12.8)	9.3 (9.5)	9.1 (9)	51 (36.5)	75 (31)	74 (30)
Percent perseverative response	15 (10)	9.2 (8.8)	9 (8)	46 (36)	73 (34)	72 (36)
Perseverative errors	10.7 (8.7)	5.8 (3.4)	5.5 (3)	59 (37)	85.6 (16)	83 (12)
Percent perseverative errors	10.3 (7)	6.7 (3)	6.5 (3)	63.6 (35)	86.7 (15)	88 (19)
Non-perseverative errors	17.6 (15.6)	7 (3.6) *	6.8 (3.3) *	48.2 (30)	76 (23) *	78 (20) *
Percent non-perseverative errors	12.2 (3.2)	7.16 (2.5) **	6.9 (2) **	44 (23)	72 (22) **	75 (19) **
Conceptual level responses	67 (6.4)	72 (12)	73 (13)	--	--	
Percent conceptual level responses	71 (9)	79 (5.6) *	80 (5.8) *	--	--	
Number of categories completed	5.9 (0.3)	6 (0)	6.1 (0.2)	--	--	
Trials to complete first categories	14.7 (4.9)	15.3 (6.7)	15 (6.5)	--	--	
Failure to maintain set	0.2 (0.4)	0.7 (1)	0.8 (0.9)	--	--	
Learning to learn	0.28 (2.4)	0 (3.19)	0 (3)			

* *p* < 0.05; ** *p* < 0.01.

**Table 5:** Results of the Test of Attentional Performance (TAP test) before and on LNAAs therapy.

Variables	T0 Ms Mean (SD)	T3 Ms Mean (SD)	T12 Ms Mean (SD)
**Vigilance**			
Time to complete the total tasks	706 (82)	614 (64) *	609 (60) *
**Sustained attention**			
Total time	727 (147)	597 (106) **	589 (98) **
*n* of errors	6 (7.7)	0.6 (0.8) **	0.8 (0.5) **
*n* of omissions	9 (9.5)	3 (2.4) *	3.2 (2.2) *

* *p* < 0.05; ** *p* < 0.01.
